# Subchondral bone remodelling and osteoarthritis

**DOI:** 10.1186/ar3713

**Published:** 2012-03-08

**Authors:** Jorge A Román-Blas, Santos Castañeda, Raquel Largo, Gabriel Herrero-Beaumont

**Affiliations:** 1 Bone and Joint Research Unit, Service of Rheumatology, IIS Fundación Jiménez Díaz, Universidad Autónoma, Madrid, Spain; 2 Department of Rheumatology, Hospital de la Princesa, IIS Princesa, Universidad Autónoma, Madrid, Spain

## 

Osteoarthritis (OA) emerges of the inharmonious functioning of joint tissues, particularly subchondral bone (SB) and articular cartilage that are two mechanically and biologically intertwined tissues [1]. Thus, biomechanical, biochemical and/or genetic alterations affecting any joint tissue may cause anomalous intra-articular stresses and subsequent tissue damage associated to a failure of repair [2]. Specific anatomical regions have been described in the bone underlying joint cartilage, including the subchondral cortical plate, subchondral trabecular bone and sub-articular bone [3]. Each region likely contributes differently to cartilage pathology. However, a lack of clear boundaries between these tissues by current imaging techniques generates some confusion in their study and thorough research will help to improve our understanding of SB properties. In addition, bone at the joint margins is markedly active since is the site of osteophyte development in OA. The close relationship between SB and joint cartilage evokes an unanswered question with valuable therapeutic implications [4]. In this context, the relationship among SB microstructure and remodeling, and cartilage destruction becomes important.

Yet, it remains controversial whether SB alterations precede the cartilage damage or they further appear during the evolution of the disease. In fact, SB remodeling abnormalities, especially increased bone turnover, have been detected early in the evolution of some forms of OA in animal models [5,6] and humans [7,8]. On the other hand, OA and systemic osteoporosis (OP) share a paradoxical relationship, being probable that high as well as low bone mass conditions result in induction and/or OA progression [4]. Interestingly, improving SB integrity showed to reduce the progression of cartilage damage in an animal model of OA preceded by OP [9]. Therefore, both bone mass phenotypes may be considered risk factors for OA initiation. The presence of other risk factors such as skeletal shape abnormalities, joint overload or obesity may have a synergistic effect for OA initiation. In addition, inflammatory mediators released by the articular cartilage may lead to SB loss by increasing bone remodeling in OA. Accordingly, OA treatment goals must consider the improvement of SB integrity. This therapeutic approach should be individualized depending on the patient BMD status and OA phenotype, and subsequently the use of drugs should also be individualized for each patient [10]. Recent findings suggest that the same drugs could be useful for treating simultaneously both processes, at least in a subgroup of patients with OA and concomitant OP.
